# Screening for low testosterone is needed for early identification and treatment of men at high risk of mortality from Covid-19

**DOI:** 10.1186/s13054-020-03086-z

**Published:** 2020-06-19

**Authors:** Simon Peter Rowland, Elizabeth O’Brien Bergin

**Affiliations:** Besins Healthcare Ireland Ltd, 16 Upper Pembroke Street, Dublin 2, Ireland

A repeated observation that disease severity and mortality rate of Covid-19 is significantly higher in male rather than female sufferers [1] has led researchers to investigate the role of sex hormones in disease progression. Recent reports of case series from China [2], Germany [3] and Italy [4] have highlighted strong associations between serum testosterone levels, inflammatory cytokines, disease progression and clinical outcomes in male Covid-19 patients, independent of patient age and comorbidities. In a cohort of 31 Italian male hospital inpatients, a significant stepwise decline in calculated free (cFT) and total testosterone (TT) levels was strongly correlated with need for escalation of care from general ward based to specialist respiratory and intensive care [4]. There was significant negative correlation between both total and free testosterone with inflammatory markers such as neutrophil count, LDH and PCT, CRP and ferritin and a positive correlation with lymphocyte count. The probability of being transferred to the ICU or dying below and above a TT level of 5 nmol/L was 14.18% [8.89–17.03] vs 0.60% [0.12–3.32] (*p* < 0.0001) and 12.40% [6.77–16.43] vs 0.39% [0.07–2.26] (*p* < 0.0001) respectively. Similar observations were made in a cohort of 45 German patients with approximately 70% having low testosterone on admission to ICU with 7 of the 9 subsequent mortalities having significantly reduced TT levels [3].

Low testosterone levels in men admitted to hospital with acute illness have previously been described in published data and have similarly been directly associated with risk of admission to intensive care and severity of disease, as measured by likelihood of development of ARDS, length of ICU stay and mortality [5]. Expert commentators have however put forward hypothesise for disease-specific processes to suggest a potentially causative effect of low testosterone on adverse clinical outcomes in Covid-19. One theory is that low testosterone levels could theoretically be detrimental because of the role of testosterone in inducing the angiotensin-converting enzyme 2 (ACE2) expression, which is an important lung protective enzyme.

Further research is needed to define the cause and effect relationship between testosterone and severe acute illness from Covid-19; however, the importance of low testosterone as a prognostic marker of severe disease is clear and as yet underrecognised in men with Covid-19. We call for wider screening of testosterone levels in men admitted to hospital with symptoms of Covid-19 as a strategy to identify those at highest risk of severe disease leading to ICU admission and mortality.

## Data Availability

Not applicable
